# Survival Analysis of Minimally Invasive Mitral Valve Surgery Versus Conventional Median Sternotomy in the United States

**DOI:** 10.7759/cureus.81859

**Published:** 2025-04-07

**Authors:** Songphol Tungjitviboonkun

**Affiliations:** 1 School of Medicine, University of California San Francisco, San Francisco, USA

**Keywords:** minimally invasive surgery, mini-mitral surgery, mini-thoracotomy, retrospective cohort study, survival analysis

## Abstract

Background: Minimally invasive mitral valve surgery (MiMVS), particularly via right mini-thoracotomy, has gained popularity as an alternative to median sternotomy, potentially reducing surgical trauma and recovery time. However, recent data on its surgical outcomes remain limited. To provide updated insights while minimizing selection bias, we analyzed elective patients undergoing mitral valve surgery, comparing MiMVS and sternotomy in terms of survival, operative times, and perioperative complications.

Methods: We conducted a single-center retrospective cohort study that included patients who underwent mitral valve surgery between 2015 and 2024. Patients were stratified into MiMVS or sternotomy groups. Kaplan-Meier survival curves and log-rank tests assessed survival, while propensity score matching (PSM) minimized selection bias.

Results: Among 422 patients (319 MiMVS, 103 sternotomy), the MiMVS group had a shorter hospital stay (5.0 vs. 8.0 days, p < 0.01) and lower postoperative bleeding (3.9% vs. 9%). Median cross-clamp and cardiopulmonary bypass (CPB) times were shorter in MiMVS (76 vs. 94 min, p < 0.01; and 114 vs. 140 min, p < 0.01, respectively). Survival analysis showed no significant difference between groups (log-rank p = 0.07) after PSM. The adjusted hazard ratio for mortality in MiMVS versus sternotomy was 0.30 (95% CI: 0.08-1.12, p = 0.07). However, mitral replacement was associated with a significantly higher mortality risk than mitral repair (HR 5.22, 95% CI: 1.26-21.61, p = 0.04). In-hospital mortality was comparable (1.9% for sternotomy vs. 0.6% for MiMVS, p = 0.25). Reoperation rates at five and 10 years were lower in MiMVS (1.7% vs. 2.1% at five years and 1.7% vs. 3.2% at 10 years).

Conclusions: While MiMVS offers advantages such as shorter hospital stays and lower postoperative bleeding rates, no statistically significant difference in overall survival was found compared to sternotomy. However, a trend toward improved survival with MiMVS was observed. Notably, mitral valve replacement was associated with a significantly higher mortality risk than mitral repair, emphasizing the importance of prioritizing repair whenever feasible.

## Introduction

Mitral valve disease is a significant cardiovascular disease that affects millions of people worldwide and accounts for approximately 15% of all deaths related to valvular heart disease [1]. The two most common forms, mitral valve regurgitation and mitral valve stenosis, can lead to heart failure if left untreated. In 2020, the crude death rate for adults with mitral valve disease in the United States was 1.6 per 100,000 [2].

Traditionally, mitral valve repair and replacement (MVR) has been performed by median sternotomy with cardiopulmonary bypass (CPB), providing direct visualization and access to the heart. However, in recent decades, minimally invasive mitral valve surgery (MiMVS) has emerged as a viable alternative to the conventional approach. There are four minimally invasive approaches to the mitral valve: (i) right mini-thoracotomy, (ii) total endoscopic approach, (iii) robotic mitral surgery, and (iv) transapical beating heart off-pump NeoChord repair [3]. However, patient selection for minimally invasive surgery remains challenging, with relative contraindications that include a poor left ventricular ejection fraction (LVEF <30%), severe right ventricular dysfunction, concomitant coronary artery bypass grafting (CABG), endocarditis, etc. [4]

This study focuses on the mini-thoracotomy approach, the most commonly performed MiMVS. This technique is performed under CPB using the Seldinger technique [5]. The heart is accessed by a lateral right thoracic incision in the third or fourth intercostal space, typically measuring around 4-5 cm. In men, the incision is located 2-3 cm lateral to the nipple, centered on the anterior axillary line, while in women it can be concealed in the submammary crease.

A previous meta-analysis, including 6,792 patients undergoing mitral valve repair, demonstrated equivalent outcomes between MiMVS and conventional sternotomy. Although MiMVS was associated with longer cross-clamp, CPB time, and operative times, it resulted in significantly shorter hospital stays, with no observed differences in long-term mortality [6]. A single-center study analyzing data from 2016 to 2021 found comparable cross-clamp time, reduced postoperative morbidity, and shorter hospital length of stay in the MiMVS group [7]. A more recent meta-analysis, pooling data from 38,106 patients, reinforced the result of shorter lengths of hospital stay without significant differences in short-term morbidity and mortality [8].

Despite these findings, the long-term survival benefit of MiMVS compared to conventional median sternotomy remains debatable. The primary aim of this study is to compare survival outcomes between MiMVS and conventional median sternotomy, utilizing data from the University of California San Francisco (UCSF) Medical Center. By assessing patient survival trends from 2015 to 2024, we aim to determine whether MiMVS offers a survival advantage over conventional median sternotomy. The secondary aims are to compare surgical outcomes such as operation time, cross-clamp time, CPB time, hospital length of stay, and perioperative outcomes including bleeding and in-hospital mortality.

## Materials and methods

Study design and data source

This single-center retrospective cohort study utilized data from the electronic medical records (EMR) of UCSF Medical Center. The study included patients who underwent mitral valve surgery between 2015 and 2024 at the Department of Cardiothoracic Surgery. EMR data were retrieved based on ICD-10-PCS codes, and operative notes were reviewed. Surgical data, including procedure type, cross-clamp time, CPB time, and postoperative diagnosis, were extracted by the authors. Additional patient characteristics such as age, sex, race, BMI, smoking status, ASA physical status, length of hospital stay, operation time, and in-hospital mortality were also collected from the EMR. Comorbidities were identified using ICD-10 codes, and death records were linked to the California Death Registry. Any discrepancies in data extraction were resolved by consensus among the authors.

Study population

Inclusion Criteria

Adult patients (≥18 years) who underwent mitral valve repair or replacement (MVR) between 2015 and 2024, procedures classified as minimally invasive or conventional median sternotomy surgery, were included.

Exclusion Criteria

Inpatient urgent consultation cases or non-elective cases were excluded from the study.

Statistical analysis

Statistical analyses were performed using STATA (StataCorp LLC, College Station, TX, USA) version 18.5. Continuous variables were compared between surgical approaches using linear regression, while categorical variables were analyzed using Fisher’s exact test. Kaplan-Meier survival curves were constructed to assess survival differences, and the log-rank test was used for comparisons. Cox proportional hazards regression was employed to estimate adjusted hazard ratios for mortality while accounting for potential confounders. To minimize selection bias, propensity score matching (PSM) was applied.

Ethical consideration

This study utilized a de-identified database, ensuring patient anonymity and confidentiality. As all data were de-identified, informed consent was not required.

## Results

Baseline characteristics

Between 2015 and 2024, a total of 542 mitral valve surgeries were performed, including 188 sternotomy and 354 mini-thoracotomy procedures. Among these, 17 (9.0%) sternotomy cases developed post-op bleeding that required one chest wash, while one (0.5%) case required two chest washes. In the mini-thoracotomy group, 11 (3.1%) cases required one re-thoracotomy, and three (0.8%) required two re-thoracotomy. Additionally, four patients with a history of median sternotomy later underwent mini-thoracotomy, while three patients with prior mini-thoracotomy underwent sternotomy. One case initially performed via mini-thoracotomy was converted to sternotomy due to uncontrolled intraoperative bleeding. After excluding non-elective cases (85 sternotomy and 35 mini-thoracotomy procedures), a total of 422 patients were included in the final analysis: 103 (24.4%) underwent conventional sternotomy, and 319 (75.6%) underwent mini-thoracotomy (Table 1). The median age of patients was 65 years, with no statistically significant difference between the two groups (p = 0.12). However, BMI was significantly lower in the mini-thoracotomy group (p < 0.001).

**Table 1:** Baseline characteristics by the surgical approach Age: Median (Min, Max); BMI: Median (Q1,Q3) Linear regression is used for continuous variables; Fisher's exact test is used for categorical variables

	Median sternotomy	Mini-thoracotomy	p-value
	N=103	N=319	
Age	63.0 (21.0,83.0)	65.0 (20.0,86.0)	0.12
BMI	25.2 (22.7,30.1)	24.1 (21.6,27.1)	<0.001
Sex			
Female	51 (49.5%)	137 (42.9%)	0.26
Male	52 (50.5%)	182 (57.1%)	
Race/Ethnicity			
Asian	23 (22.3%)	55 (17.2%)	0.22
Black or African American	6 (5.8%)	11 (3.4%)	
Latinx	11 (10.7%)	30 (9.4%)	
Multi-Race/Ethnicity	5 (4.9%)	12 (3.8%)	
Native American or Alaska Native	0 (0.0%)	1 (0.3%)	
Native Hawaiian or Other Pacific Islander	2 (1.9%)	0 (0.0%)	
Other	1 (1.0%)	6 (1.9%)	
Southwest Asian and North African	3 (2.9%)	6 (1.9%)	
Unknown/Declined	0 (0.0%)	4 (1.3%)	
White	52 (50.5%)	194 (60.8%)	
HIV			
Negative	101 (98.1%)	306 (95.9%)	0.54
Positive	2 (1.9%)	13 (4.1%)	
Diabetes			
No	83 (80.6%)	276 (86.5%)	0.15
Yes	20 (19.4%)	43 (13.5%)	
Cardiovascular disease			
No	94 (91.3%)	272 (85.3%)	0.13
Yes	9 (8.7%)	47 (14.7%)	
Cerebrovascular disease			
No	99 (96.1%)	303 (95.0%)	0.79
Yes	4 (3.9%)	16 (5.0%)	
COPD			
No	90 (87.4%)	277 (86.8%)	1.00
Yes	13 (12.6%)	42 (13.2%)	
Smoking status			
Every Day	3 (2.9%)	0 (0.0%)	0.02
Former	40 (38.8%)	108 (33.9%)	
Never	59 (57.3%)	207 (64.9%)	
Some Days	0 (0.0%)	3 (0.9%)	
Unknown	1 (1.0%)	1 (0.3%)	
ASA Physical status			
*Unspecified	1 (1.0%)	0 (0.0%)	0.00
1	0 (0.0%)	1 (0.3%)	
2	4 (3.9%)	41 (12.9%)	
3	67 (65.0%)	215 (67.4%)	
4	31 (30.1%)	62 (19.4%)	

Surgical outcomes

The surgical outcomes are summarized in Table 2. Patients who underwent mini-thoracotomy had significantly shorter operative time (3.9 hours vs. 4.5 hours, p < 0.001), cross-clamp times (76 minutes vs. 94 minutes, p < 0.001), and CPB times (114 minutes vs. 140 minutes, p = 0.001) compared to the sternotomy group. Regarding disease etiology, mitral regurgitation was more common in the mini-thoracotomy group (92.5% vs. 76.7%, p < 0.001), while mitral stenosis (15.5% vs. 4.7%) and prosthetic valve degeneration (6.8% vs. 0%) were more frequent in the sternotomy group. Mitral repair was performed more frequently in mini-thoracotomy (75.6% vs. 33.3%, p < 0.001), whereas mitral valve replacement was more common in sternotomy (66.7% vs. 24.4%). Mini-thoracotomy patients had a significantly shorter median hospital stay (5 vs. 8 days, p<0.001). The in-hospital mortality rate was low in both groups, with no statistically significant difference (p = 0.25).

**Table 2:** Outcomes by the surgical approach Length of stay, cross-clamp time, cardiopulmonary bypass time = Median (Q1, Q3) Linear regression is used for continuous variables; Fisher's exact test is used for categorical variables

	Median sternotomy	Mini-thoracotomy	p-value
	N=103	N=319	
Length of stay (days)	8.0 (6.0,13.0)	5.0 (4.0,7.0)	<0.001
Operation time (hours)	4.5 (4.0,5.4)	3.9 (3.4,4.5)	<0.001
Cross-clamp time	94.0 (85.0,136.0)	76.0 (65.0,89.0)	<0.001
Cardiopulmonary bypass time	140.0 (118.0,185.0)	114.0 (102.0,136.0)	0.00
Disease etiology			
Mitral regurgitation/insufficiency	79 (76.7%)	295 (92.5%)	<0.001
Mitral stenosis	16 (15.5%)	15 (4.7%)	
Mitral regurgitation/stenosis	1 (1.0%)	6 (1.9%)	
Prosthetic valve degeneration	7 (6.8%)	0 (0.0%)	
Atrial mass	0 (0.0%)	3 (0.9%)	
Procedure type			
Mitral repair	34 (33.3%)	238 (75.6%)	<0.001
Mitral replacement	68 (66.7%)	77 (24.4%)	
Endocarditis			
No	101 (98.1%)	318 (99.7%)	0.15
Yes	2 (1.9%)	1 (0.3%)	
CABG			
No	99 (96.1%)	319 (100.0%)	0.00
Yes	4 (3.9%)	0 (0.0%)	
In-hospital mortality			
No	101 (98.1%)	317 (99.4%)	0.25
Yes	2 (1.9%)	2 (0.6%)	

**Figure 1:**
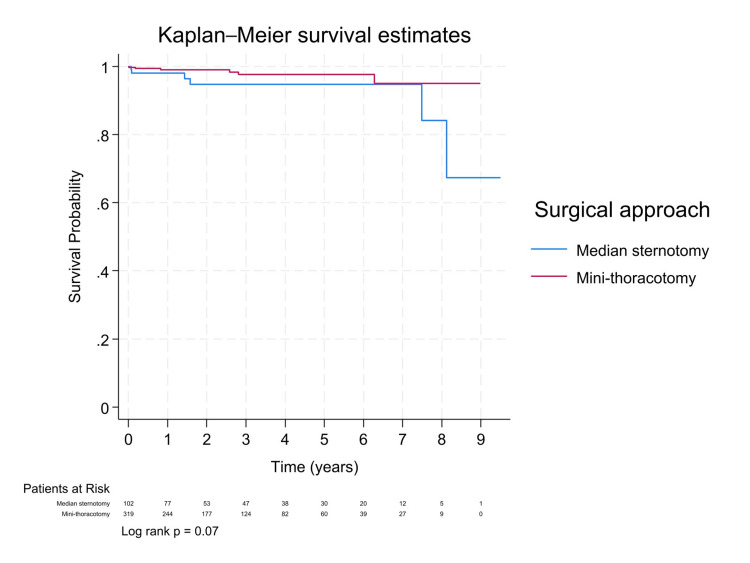
Kaplan-Meier survival curves by the surgical approach

**Figure 2:**
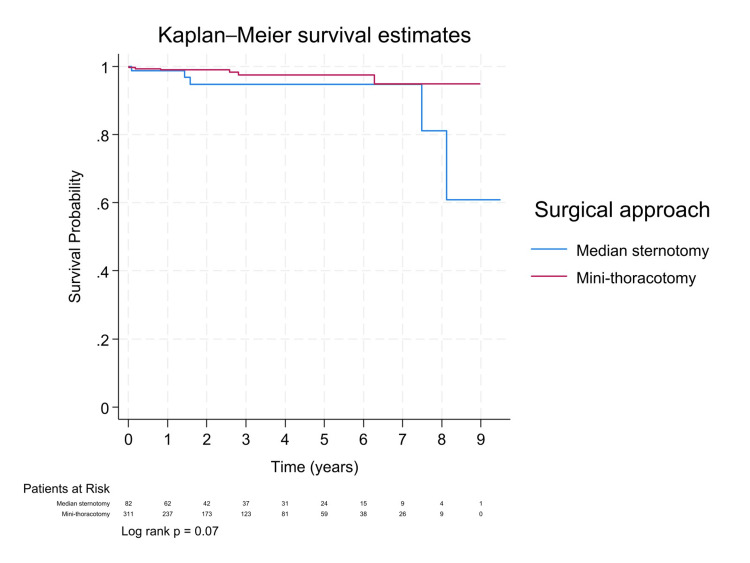
Kaplan-Meier survival curves by surgical approach after PSM PSM: Propensity score matching

**Figure 3:**
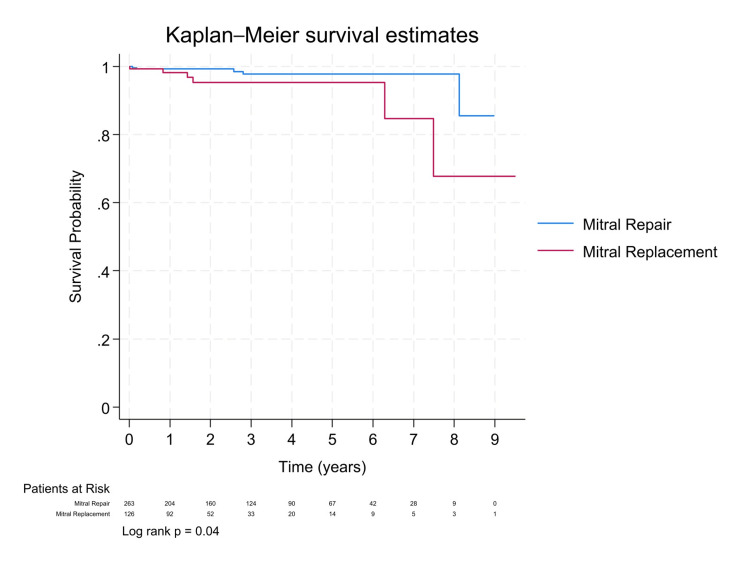
Kaplan-Meier survival curves by procedure type after PSM PSM: Propensity score matching

At a median follow-up of 1.5 years, Kaplan-Meier survival curves comparing MiMVS and sternotomy showed no statistically significant difference (log-rank p = 0.07; Figure 1). Cox proportional hazards analysis indicated a hazard ratio of 0.36 (95% CI: 0.09-1.38) for mini-thoracotomy, suggesting a potential survival benefit. After propensity score matching, the trend favoring MiMVS persisted (Figure 2), with an even lower hazard ratio (HR: 0.30, 95% CI: 0.08-1.12; Table 3), supporting a possible benefit despite limited power. A separate analysis comparing mitral valve repair and replacement showed that replacement was associated with significantly higher mortality (HR: 5.22, 95% CI: 1.26-21.61; log-rank p = 0.04; Figure 3), underscoring the importance of repair whenever feasible. Additionally, COPD and male sex were significant predictors of higher mortality in both unadjusted and propensity-matched models (Table 3).

**Table 3:** Cox proportional hazard ratio by surgical approach after propensity score matching Results are estimates with a 95% confidence interval

	Hazard Ratio	95% CI
Surgical approach		
Mini-thoracotomy	0.30	(0.08, 1.12)
Age	1.03	(0.97, 1.08)
BMI	0.94	(0.80, 1.10)
Sex	10.51	(1.24, 88.88)
HIV	4.68	(0.62, 35.49)
Diabetes	1.68	(0.37, 7.67)
Cardiovascular disease	0.41	(0.05, 3.69)
Cerebrovascular disease	0.00	-
COPD	4.84	(1.18, 19.85)
Smoking status	0.58	(0.31, 1.08)
Number of observations	393	

In-hospital mortality was similar between the two approaches (1.9% for sternotomy vs. 0.6% for mini-thoracotomy, p = 0.25).

**Figure 4:**
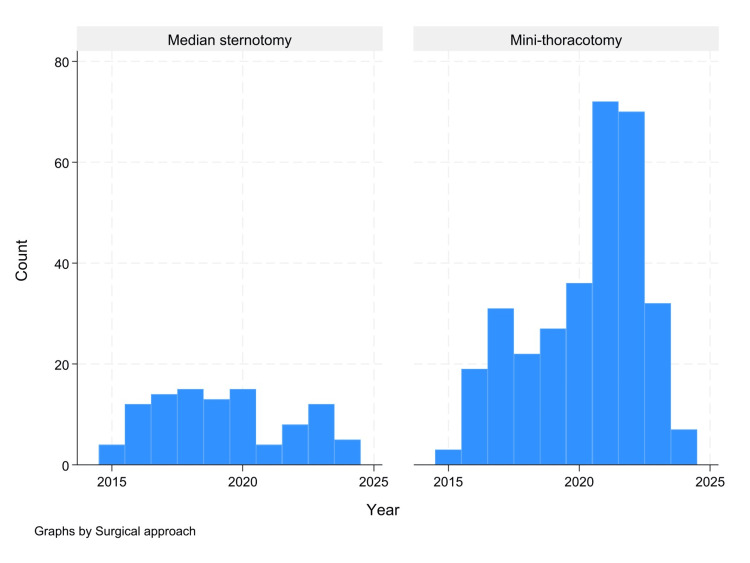
Histogram shows the number of mitral valve surgeries conducted per year between 2015 and 2024

Our data also show a rising trend in MiMVS use, with a dramatic increase in procedures after 2016 (Figure 4).

## Discussion

Our study provides key insights into MiMVS via mini-thoracotomy compared to conventional sternotomy, with a focus on perioperative outcomes, long-term survival, and reoperation rates.

To minimize selection bias, we included only elective surgical patients and excluded inpatients, who were generally sicker and more likely to undergo conventional sternotomy. Including these patients could have skewed results, likely increasing the hazard ratio for mortality in the sternotomy group.

Unlike previous studies that reported longer cross-clamp and CPB times in the mini-thoracotomy approach [6,9,10], our findings showed significantly shorter operative time, cross-clamp time, and CPB time compared to sternotomy. Additionally, our cross-clamp and CPB times were shorter than those reported in 2014, where the mean durations were 99 and 153 minutes, respectively [11]. This improvement likely reflects increased surgical experience, refined techniques, and advancements in surgical technology. Consistent with prior research, our study confirmed that MiMVS was associated with a shorter hospital length of stay [7,9,12-14].

Previous studies have also reported no significant differences in 30-day complications, long-term mortality [5,7,9], or recurrent ≥ grade 2+ mitral regurgitation between the two approaches [11]. Reoperation rates due to recurrent mitral regurgitation in our study were slightly lower than previously reported. Among 188 patients who initially underwent sternotomy, four patients (2.1%) required reoperation within five years and six patients (3.2%) within 10 years. In contrast, of 354 patients who had an initial mini-thoracotomy, six patients (1.7%) required reoperation within five years and 10 years. These rates compare favorably to prior findings, which reported freedom from mitral valve reoperation at 96.8% and 93.8% at 5 and 10 years, respectively [11].

Our data also revealed a rising trend in MiMVS adoption, with a dramatic increase in procedures after 2016. As minimally invasive approaches have gained popularity, surgeons have refined their techniques, leading to greater efficiency.

Although our study did not find a statistically significant survival benefit for mini-thoracotomy, we observed a trend toward improved survival, contradicting a 2018 study that suggested a trend favoring sternotomy [10]. However, neither study reached statistical significance. Notably, our study is the first to report that mitral valve replacement carries a higher mortality risk than mitral valve repair, reinforcing the importance of repair-focused approaches whenever feasible.

Although this study addresses selection bias using PSM, other limitations persist. First, the retrospective design inherently carries the risk of bias due to its reliance on historical data, which may contain inaccuracies. Second, there could be unmeasured biases that were not included in the study. Finally, the study was conducted at a single medical school tertiary-care facility, which limits the generalizability of the findings to broader populations.

## Conclusions

Using data from recent decades, our study underscores the advantages of minimally invasive mitral valve surgery via mini-thoracotomy, demonstrating shorter operative and cross-clamp times, reduced hospital stays, and lower postoperative bleeding rates compared to conventional sternotomy. While no statistically significant survival advantage was observed, a trend toward improved survival with mini-thoracotomy suggests potential long-term benefits. Most importantly, our findings highlight the significantly higher mortality risk associated with mitral valve replacement compared to mitral valve repair, emphasizing the need to prioritize repair whenever feasible. Additionally, the increasing adoption of MiMVS over time reflects growing surgical expertise and advancements in technology, which may further improve outcomes in the future. These insights contribute to the ongoing evolution of mitral valve surgery, reinforcing the importance of refining patient selection criteria and surgical techniques to maximize the benefits of minimally invasive approaches. Future studies should focus on long-term survival, durability of repair, and optimal strategies to expand the use of minimally invasive techniques while maintaining procedural safety and efficacy.
