# Correction: Survival Analysis of Minimally Invasive Mitral Valve Surgery Versus Conventional Median Sternotomy in the United States

**DOI:** 10.7759/cureus.c389

**Published:** 2026-01-07

**Authors:** Songphol Tungjitviboonkun

**Affiliations:** 1 School of Medicine, University of California San Francisco, San Francisco, USA

This article has been corrected at the request of the authors to remove Pongsaya Saipia from the author list. 

